# Abrogated Inflammatory Response Promotes Neurogenesis in a Murine Model of Japanese Encephalitis

**DOI:** 10.1371/journal.pone.0017225

**Published:** 2011-03-03

**Authors:** Sulagna Das, Kallol Dutta, Kanhaiya Lal Kumawat, Ayan Ghoshal, Dwaipayan Adhya, Anirban Basu

**Affiliations:** National Brain Research Centre, Manesar, Haryana, India; University of Nebraska Medical Center, United States of America

## Abstract

**Background:**

Japanese encephalitis virus (JEV) induces neuroinflammation with typical features of viral encephalitis, including inflammatory cell infiltration, activation of microglia, and neuronal degeneration. The detrimental effects of inflammation on neurogenesis have been reported in various models of acute and chronic inflammation. We investigated whether JEV-induced inflammation has similar adverse effects on neurogenesis and whether those effects can be reversed using an anti-inflammatory compound minocycline.

**Methodology/Principal Findings:**

Here, using *in vitro* studies and mouse models, we observed that an acute inflammatory milieu is created in the subventricular neurogenic niche following Japanese encephalitis (JE) and a resultant impairment in neurogenesis occurs, which can be reversed with minocycline treatment. Immunohistological studies showed that proliferating cells were replenished and the population of migrating neuroblasts was restored in the niche following minocycline treatment. *In vitro*, we checked for the efficacy of minocycline as an anti-inflammatory compound and cytokine bead array showed that production of cyto/chemokines decreased in JEV-activated BV2 cells. Furthermore, mouse neurospheres grown in the conditioned media from JEV-activated microglia exhibit arrest in both proliferation and differentiation of the spheres compared to conditioned media from control microglia. These effects were completely reversed when conditioned media from JEV-activated and minocycline treated microglia was used.

**Conclusion/Significance:**

This study provides conclusive evidence that JEV-activated microglia and the resultant inflammatory molecules are anti-proliferative and anti-neurogenic for NSPCs growth and development, and therefore contribute to the viral neuropathogenesis. The role of minocycline in restoring neurogenesis may implicate enhanced neuronal repair and attenuation of the neuropsychiatric sequelae in JE survivors.

## Introduction

The host response to infection is central to the effective control and ultimate clearance of invading pathogens or removal of infected cells. The response to virus infections of the CNS is characterised by microglial activation, significant recruitment and extravasation of peripheral immune cells to the sites of virus replication in the brain [1], [2]. The ensuing neuroinflammation is a hallmark of most neurotropic viruses and correlates with the neuropathological outcome of the virus infection. Besides the direct involvement of the virus in destruction of brain tissue, indirect/bystander mode of CNS damage due to microglial activation and resultant neuroinflammatory changes has been observed in a number of CNS infections [3], [4], [5].

Japanese encephalitis virus (JEV) belonging to the family *Flaviviridae*, causes acute zoonotic infection that commonly affects children and leads to fatalities in 30% of the cases [6], [7]. In 50% of the survivors of Japanese encephalitis (JE), neuropsychiatric sequelae ranging from speech and motor problems, movement disorders, and behavioural abnormalities have been reported [8], [9]. Besides causing massive neuronal death [10], JEV induces global inflammation which includes microglial activation and production of pro-inflammatory mediators. We have already shown the deleterious effects of microglial activation and inflammatory molecule production on neuronal health. The various soluble mediators released upon infection of microglia and/or infiltrating macrophages include cytokines, chemokines and other bioactive molecules including ROS and NO [3].

Recent years have demonstrated that microglia and ensuing inflammation are critical regulators of post-natal/adult neurogenesis [11]. Neurogenesis continues beyond embryonic life, albeit at a slow rate in two specific neurogenic areas, the subventricular zone (SVZ) and the subgranular zone (SGZ) of the hippocampus [12]. Both these areas are populated by self-renewing, multipotential cells referred to as neural stem/progenitor cells (NSPCs) [13]. The dual role of microglia in modulating neurogenesis has been postulated depending on the state/mode of its activation. Detrimental effects of activated microglia has been observed on the survival of newly formed hippocampal neurons *in vivo* [14] as well as *in vitro* when NSPCs were grown in conditioned media from LPS-activated BV2 cells [15]. The key mechanism by which these adverse effects of microglia on neurogenesis are brought about is via the release of pro-inflammatory mediators such as IL-1β, IL-6, IFN-γ, TNF-α, and IL-8 from activated microglia, which are anti-neurogenic [16], [17]. While IL-1β and IL-6 have been strongly implicated in decreased NSPC proliferation and neurogenesis [18], [19], others like TNF-α exert dual effects depending on the receptor types involved. Action via TNFR1 suppresses NSPC proliferation in adult hippocampus both in normal and diseased brain, whereas TNFR2 improves the proliferation and survival of newly formed hippocampal neurons [20]. It has now been suggested that microglial activation is not pro- or anti-neurogenic *per se*, but the resultant effect on neurogenesis is dependent on the balance between secreted pro- and anti-inflammatory molecules [21].

The effect of virus-induced inflammation on neurogenesis has been best illustrated in case of HIV-1 infections. The various chemokines released upon HIV-1 infection acting via their chemokine receptors on the NSPCs induce quiescence in these cells [22] and also results in deficits in neurogenesis culminating in impairment of development, motor and cognitive functions [23]. The HIV-1 infected human monocyte derived macrophages (MDM) increased the proliferation of human cortical NSPC cultures, but inhibited their neuronal differentiation to β-III tubulin positive cells [24], [25].

Anti-inflammatory compounds have been shown to be effective in restoring neurogenesis in a number of inflammatory conditions like cranial irradiation, epilepsy, and ischemic stroke [26], [14], [27], [28]. Non steroidal anti-inflammatory drugs (NSAIDs), which are therapeutic agents of first choice for the treatment of inflammation, in both chronic and acute neuropathologies, have also been shown to promote neurogenesis by attenuating microgliosis and inflammation [29]. The second generation antibiotic minocycline with its potent anti-inflammatory properties have been shown to be beneficial for neurogenesis in a number of injury models by reducing microglial activation. Minocycline promoted the survival of newly generated neurons as well as functional improvement in hippocampal spatial memory tasks in stroke models like MCAO in rats [28].

Previous findings from our laboratory have already established minocycline as a potent anti-inflammatory compound, which alleviates the symptoms of JE [30]. Besides, minocycline confers protection to these animals and significantly improves their survival. Furthermore, we have also reported that post-natal SVZ neurogenesis is severely impaired in JEV-infected animals [31]. Besides the direct effect of the virus on the development of NSPCs, here we wanted to investigate how JEV-induced inflammation modulates the fate of these cells, and whether the use of the anti-inflammatory compound minocycline reverses these effects.

## Materials and Methods

### Ethics Statement

All animals were handled in strict accordance with good animal practice as defined by Institutional Animal and Ethics Committee (IAEC) of National Brain Research Centre. The animal experiment protocol approval numbers are NBRC/IAEC/2008/41 and NBRC/IAEC/28/2005. Animals were handled in strict accordance with good animal practice as defined by the Committee for the Purpose of Control and Supervision of Experiments on Animals (CPCSEA), Ministry of Environment and Forestry, Government of India. All animal studies were approved by the IAEC of National Brain Research Centre.

### Virus generation and titration

The GP78 strain of JEV was propagated in suckling BALB/c mice and their brains were harvested when symptoms of sickness were observed. A 10% tissue suspension was made in MEM, followed by centrifugation at 10,000×g and finally filtered through a 0.22 µm sterile filter. The titration of virus particles was done by plaque formation using PS (Porcine stable Kidney) cell line as described before and plaques were counted [10], [32].

### Virus infection of animals and minocycline administration

Adult animals (4–6 weeks), were randomly assigned into 3 groups: the Control group (Control); the JEV-infected group (JEV); and the JEV-infected and minocycline treated group (JEV+M). BALB/c mice of either sex were injected with 3×10^5^ p.f.u of JEV strain GP78 through tail-vein (intravenous). Control animals received PBS. Minocycline (Sigma) at a dose of 45 mg/kg body weight was administered intraperitoneally next day after JEV infection and continued for 6 days [30]. From 5^th^ day onwards animals started to show symptoms of JE including restriction of movements, limb paralysis, poor pain response, whole body tremor, piloerection and hind limb paralysis. On the ninth day post infection all animals succumbed to death. Sets of five mice from Control and JEV-infected group were sacrificed at 9^th^ day post infection time point either for tissue, protein or RNA. The JEV+M group were sacrificed 15 days after the JEV-infected animals succumbed to infection.

### Viral titration

The SVZ from individual animals from all 3 different groups was dissected out after sacrificing. A 10% tissue suspension was made in MEM (minimum essential medium), followed by centrifugation at 10,000×g and finally filtered through a 0.22 µ sterile filter [33]. JEV was titrated by plaque formation on Vero cell monolayer. Vero cells were seeded in six-well plates to form semi-confluent monolayer in about 18 h. Cell monolayer were inoculated with 10-fold serial dilutions of tissue suspension made in MEM containing 1% FBS and incubated for 1 h at 37°C with occasional shaking. The inoculum was removed by aspiration and the monolayers were overlaid with MEM containing 4% FBS, 1% low-melting-point agarose and a cocktail of antibiotic–antimycotic solution (Gibco, Invitrogen Corporation, Grassland, NY, USA) containing penicillin, streptomycin, and amphotericin B. Plates were incubated at 37° C for 72–96 h until plaques became visible. To allow counting of the plaques, the cell monolayer was stained with crystal violet after fixing the cells with 10% paraformaldehyde.

### BrdU labelling

One set of animals (n = 3) from each group received intraperitonal-injections of BrdU (50 mg/kg body weight; Sigma) twice daily for 5 days. Control and JEV-infected animals received BrdU injections from 5^th^ day p.i. onwards, while animals from JEV+M received BrdU from 20^th^ day p.i. onwards. All animals were sacrificed 6 h after the last BrdU injection.

### Cell culture

Mouse microglial cell line BV2 was a kind gift from Dr. Steve Levison, University of Medicine and Dentistry, New Jersey, USA. The cell line was grown at 37°C in DMEM supplemented with 5% sodium bicarbonate, and 10% FBS and 1% penicillin/streptomycin solution. All reagents for cell culture were obtained from Sigma, St. Louis, USA unless otherwise mentioned.

Mouse neurospheres were cultured from post-natal day 7 BALB/c mouse pups as described earlier [33]. Briefly, the SVZ was dissected out aseptically in PGM buffer (phosphate buffer with 1 mM MgCl_2_ and 0.6% glucose) and then enzymatically dissociated in a solution of 2 mg/ml Papain with 50 µg/ml DNaseI at 37°C for 10 min. Following neutralization and washes with DMEM containing 10% FBS, the cell pellet was resuspended DMEM- F12 containing B27 supplement and 50 µg/ml gentamycin (all from Gibco, Carlsbad, CA). The suspension was passed through 40 µm screen and then centrifuged at 300×g for 6 min. The cells were plated at density of 3×10^4^ cells/cm^2^ in DMEM F12 containing B27 and gentamycin, supplemented with 20 mg/ml EGF (Epidermal Growth Factor) and 10 mg/ml FGF (Fibroblast Growth Factor; R&D Systems, Minneapolis, MN). Fresh media was added after every 2 days. All *in vitro* experiments with neurospheres were carried out after minimum 2 passages and under cell density of 1.5×10^6^ cells/100 mm in petridishes.

### Differentiation of neurospheres/NSPCs

Control and BV2-CM treated neurospheres after 3 days in culture were dissociated into single cells using Accutase for 7–8 min at 37°C. DMEM-F12 media containing B27 supplements (Gibco, USA) was added to the cells and centrifuged to remove Accutase. The cell pellet was resuspended in Neurobasal media containing N2 and B27 supplements, 2 mM Glutamax and gentamycin (all from Gibco), and then triturated very gently. Cell counting was performed and plated in poly-D-lysine (PDL, Sigma) coated chamber slides or 60 mm petridishes for adhering and differentiation for 2 days. After every 2 days, 50% of media was replaced with fresh differentiation media.

### Treatment and Infection of cells

BV2 cell line was plated in 90 mm petridishes in triplicate for 3 experimental conditions- Control (BV2-C), JEV-infected (BV2-JEV) and JEV-infected and minocycline treated (BV2-JEV+M). After 24 h in DMEM with 10% serum, the cells were switched to serum free media for 6 h. BV2 cells were then adsorbed with either live JEV (MOI = 5) or mock-infected with equal volumes of sterile 1× PBS, for 1 h. After adsorption, unbound viruses were removed by gentle washing with PBS. Fresh serum free DMEM-F12 media was added to the cells. Minocycline treatment (20 µM) was done for 2 h prior to JEV infection, and then for 6 h following infection. After 6 h p.i., cells were washed with PBS and replaced with fresh DMEM-F12 in all the conditions. Finally at 12 h p.i., the media from BV2 cells was collected, centrifuged, and the supernatant collected was regarded as the microglia-conditioned media (CM) and stored at −30°C until further use. Cells were processed for immunoblot analysis as described later in materials and methods.

Mouse neurospheres after two passages were grown either as control or in the presence of 50% of conditioned media from BV2-C, BV2-JEV and BV2-JEV+M conditions. After 3 days of growth, neurospheres from 4 different conditions were either mechanically dissociated and plated onto PDL-coated Petri dishes for differentiation, or collected for cell cycle analysis or for western blotting.

### Immunocytochemistry

Single cells suspensions of NSPCs grown on PDL-coated chamber slides for 2 days were washed once with PBS and fixed in 4% formaldehyde for 20 min at RT. Following PBS washes, blocking was done for 1.5 h at RT in blocking solution containing 5% serum/BSA with 0.1% Triton-X. Primary antibodies were prepared in 2% serum/BSA in 1× PBS and the cells were kept overnight in a humidified chamber at 4°C. Antibodies against the markers for neurons- β-III tubulin (1∶1000, Promega, Madison, USA) and astrocytes-GFAP (1∶500, Dako, Glostrup, Denmark) were used. After PBS washes, the corresponding secondary antibodies were added, either FITC labeled (1∶300, Vector Labs, Burlingame, CA) or Alexa 594 labeled (1∶1000, Molecular Probes, Oregon, USA), and then mounted with Vectashield containing DAPI (Vector Labs). Images were captured using Zeiss Apotome microscope.

### Immunohistochemistry

Brains from all animal groups were sectioned in a cryostat and cryosections (20 µ) were stored in −80°C until use, as described before [34]. Serial sections from adult BALB/c mouse brains containing the SVZ were used for immunohistochemistry. Antigen retrieval was done on the section using Antigen Unmasking Solution (Vector Labs) at 70°C for 1 hour. The sections were brought to room temperature, washed with PBS and then quenched using 3% H_2_O_2_ solution prepared in PBS. Following washes with PBS, permeabilization using 6 N HCl for 7 min was done, which were then neutralised with 0.1 M Borate buffer (pH 8.5) [31]. The sections were then washed with PBS and then incubated in blocking solution (5% BSA in PBS) for 2 hours. Primary antibodies against Ki-67 (1∶2000; Novocastra), PCNA (1∶2000; Cell Signalling), BrdU (1∶250; Sigma) were added and left overnight in a humidified chamber at 4°C. Following extensive washes with PBS, respective biotin-conjugated secondary antibodies were added to the sections in PBS for 1.5 h at RT. ABC Reagent (Vector, prepared 30 min in advance of use) was next added to the sections for 1 h at RT. After final washes with PBS, sections were developed using Peroxidase Substrate Kit DAB (Vector) according to manufacturer's instructions.

Fluorescence immunohistochemistry was also done on the sections for the following antibodies: anti-CD3 (1∶100; Chemicon), anti-CD11b (1∶100; Chemicon), anti-Ly6/c (1∶100; BD Biosciences) and anti-DCX (1∶750; Chemicon). However, in this case, the quenching and permeabilization step were not performed. Double immunohistochemistry for BrdU and DCX was also performed as described earlier [31].

### Immunoblotting

The SVZ from individual animals from all 3 different groups was dissected out and processed for protein isolation [3]. Twenty microgram of each sample was electrophoresed and immunoblotting was done with antibodies against iNOS (Chemicon), Cox-2 (Chemicon), PCNA, DCX, and PSD-95 (all from Cell Signaling) and against JEV non-structural (NS) proteins 3 and 5 (kind gifts from Dr Chun-Jung Chen, Taichung Veterans General Hospital, Taiwan). Respective HRP-secondary antibodies were added and blots were developed using Chemiluminescence reagent from Millipore in Syngene Gel Documentation System (Cambridge, UK). Images were captured using GeneSnap software and the densitometric analysis for the blots was done with GeneTools software (both softwares from Syngene). Similarly, cell lysates were prepared from mouse neurospheres grown in CM from BV2 cells. Forty micrograms of protein was electrophoresed and western blotting for p21, p53, p107 (all from Santa Cruz, CA, USA), p27 and cyclin D (Abcam, Cambridge, UK), was performed. The blots were stripped and reprobed with anti-β-tubulin or anti-β-actin to determine equivalent loading of samples. Densitometric analysis was done as before.

### RNA extraction and quantitative Real Time PCR (qRT-PCR)

Total RNA was isolated from differentiated NSPCs either control or CM treated after 2 days of induction of differentiation using RNAeasy Mini Kit (Qiagen, Hamburg, Germany). Random hexamer primers were used for cDNA synthesis using Advantage RT-PCR (Clontech, Mountain View, CA, USA). The forward and reverse primers for β-III tubulin (Genebank Acc No. NM_023279.2) were 5′-TGGACAGTGTTCGGTCTGG-3′ and 5′-CCTCCGTATAGTGCCCTTTGG-3′ respectively. 500 ng of cDNA was used as a template for performing qRT-PCR using SYBR Green Supermix (Bio-Rad, Hercules, CA) on ABI Prism 7700 sequence detection system (Applied Biosystems, Foster City, CA, USA). The conditions for real time PCR were as follows: 95°C for 3 min (1 cycle), 94°C for 20 s, 55°C for 30 s, and 72°C for 40 s (40 cycles). The dissociation curves were generated to check for the specificity of primer annealing to the template. The results were analyzed using the iCycler Thermal Cycler Software (Applied Biosystems) and normalized with those from 18S rRNA internal control [33].

### Cytokine Bead Array (CBA)

Different brain areas of adult BALB/c mice from the different experimental conditions were dissected, namely cortex, hippocampus, SVZ and striatum. Tissue homogenates were prepared and protein was isolated. CBA was performed to quantitatively measure the cytokine levels in each of the samples according to previously described protocol [34]. Briefly, 50 µl of bead mix from Mouse Inflammation CBA kit (BD Biosciences, San Jose, CA, USA) and 50 µl of tissue protein were incubated together for 2 h at RT in dark. The beads were then washed and resuspended in 300 µl of Wash buffer and acquired using Cell Quest Pro Software in FACS Calibur (Becton Dickinson, San Diego, CA). Analysis was done using BD CBA software from standard curves of the respective cytokines [33]. Data were represented in terms of fold change w.r.t control animals. CBA was also performed using the CM from BV2 cells, and the concentration of various cytokines in terms of pg/ml has been indicated.

### Cell cycle analysis

Neurospheres grown under different conditions were dissociated into single cells using Accutase and washed twice with PBS containing 1% BSA. Cells were then fixed with 70% ethanol for 1 h at 4°C, and then stored at −30°C [35]. The fixed cells were washed in PBS, resuspended in propidium iodide(PI)/RNase staining buffer (BD Biosciences) for 15 min at RT in dark and flow cytometric analysis of 10^6^ cells were carried out using Cell Quest program on FACS Calibur (Becton Dickinson). The linearity and doublet discrimination capacity of the instrument was tested using DNA QC particles (BD Biosciences). The percentage of cells in the G1, S, and G2/M phases of the cell cycle was analyzed with Cell Quest Program.

### Statistical Analysis

All the experiments performed and the data generated were analyzed statistically using paired and un-paired one-tailed Student's t-test. A statistical p value upto 0.05 was considered significant.

## Results

### Enhanced production of pro-inflammatory cyto/chemokines in different areas of adult JEV infection is reduced by minocycline treatment

**Figure 1:**
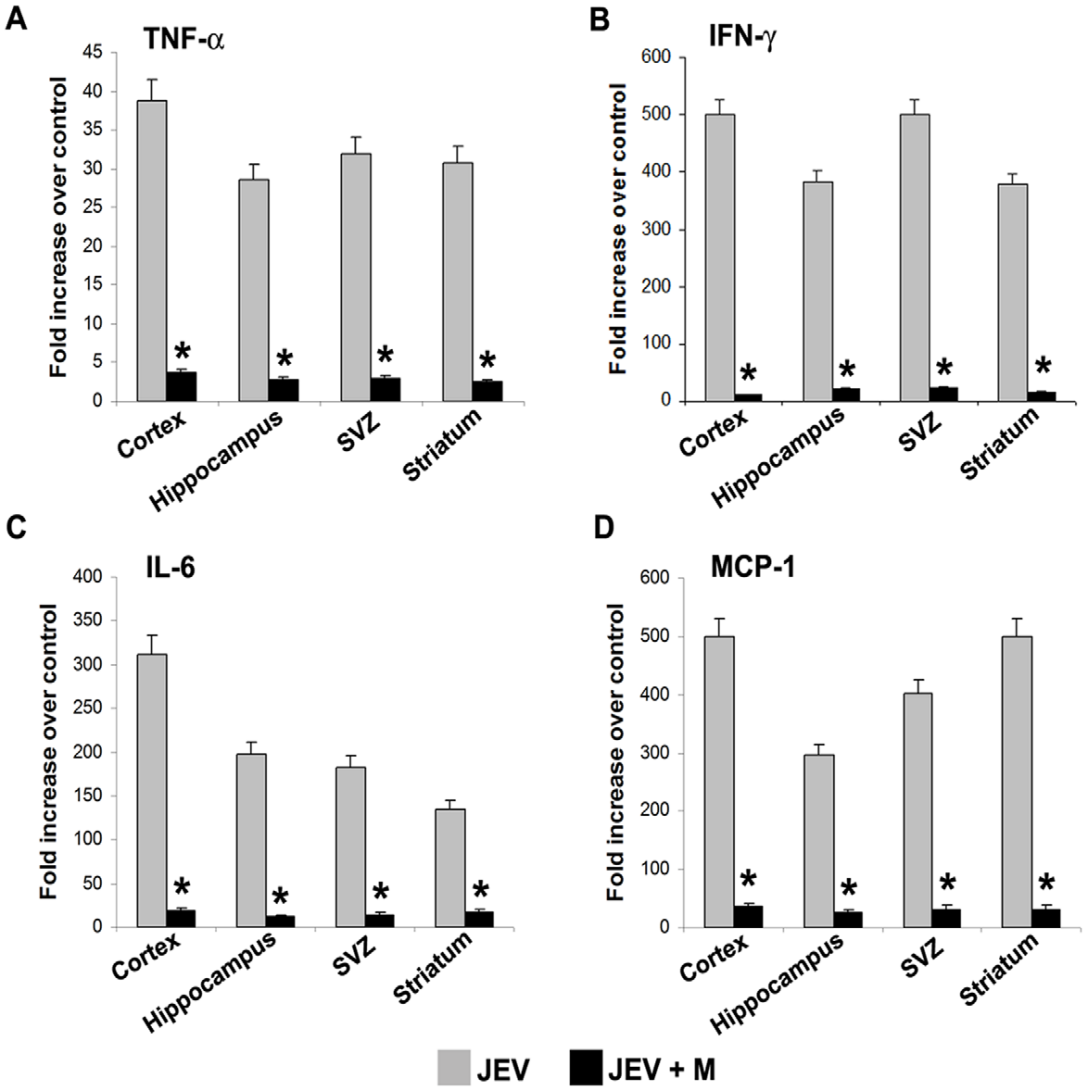
Decreased production of proinflammatory cyto/chemokines across various regions of the adult JEV-infected brain by minocycline administration. Protein isolated from cortex, hippocampus, SVZ and striatum of Control, JEV-infected and JEV+M animals were analyzed using CBA. The graphs depict the fold increase in TNF-α (**A**), IFN-γ (**B**), IL-6 (**C**) and MCP-1 (**D**) levels in the JEV-infected and JEV+M treated brain areas compared to those from control brain. A profound increase in the cytokine level of TNF-α (**A**), IFN-γ (**B**), IL-6 (**C**), and MCP-1 (**D**) was observed in all the areas of JEV-infected brain, which was dramatically reduced upon minocycline administration in JEV+M animals. (Values represent means ± SEM from five animals in each group; ^*^ significant change from JEV, p<0.01).

Different areas of brain, namely cortex, SVZ, hippocampus and striatum were dissected out from each of the 3 groups of adult animals-Control, JEV and JEV+M and CBA was performed. The production of all the cyto/chemokines-TNF-α, IFN-γ, IL-6, MCP-1 increased multifold in JEV-infected brains compared to control, which was significantly reduced upon minocycline treatment in JEV+M group (Fig. 1). Interestingly, unlike the cytokine profile of the different areas from the infected BALB/c mouse pups [3], the adult cortex show maximum increase of all the cyto/chemokines compared to other brain areas.

TNF-α showed significant 38-fold and 32-fold increases in the cortex and SVZ respectively from JEV-infected animals, which was significantly reduced in JEV+M group (Fig. 1A) (p<0.01). While IFN-γ levels increased to approximately 500 fold above control in both cortex and SVZ from infected animals, JEV-infected hippocampus and striatum showed increase to around 380 fold above control (Fig. 1B). Elevated levels of IL-6 was detected in the infected cortex at 320 fold above control, but lower levels at almost 180 fold above control was detected in both the infected neurogenic areas, SVZ and hippocampus (Fig. 1C). In case of chemokine MCP-1, a dramatic 500-fold increase above control was observed in both cortex and striatum from JEV-infected animals. MCP-1 levels in SVZ also demonstrated 400-fold increase over control, which is significantly higher than the levels observed in hippocampus at 280-fold over control (Fig. 1D) (p<0.05). Thus, between the two neurogenic areas, higher concentration of cyto/chemokines was detected in the SVZ compared to the hippocampus.

### Increased localisation of immune cells and production of inflammatory mediators in adult JEV-infected brain is diminished by minocycline treatment

**Figure 2:**
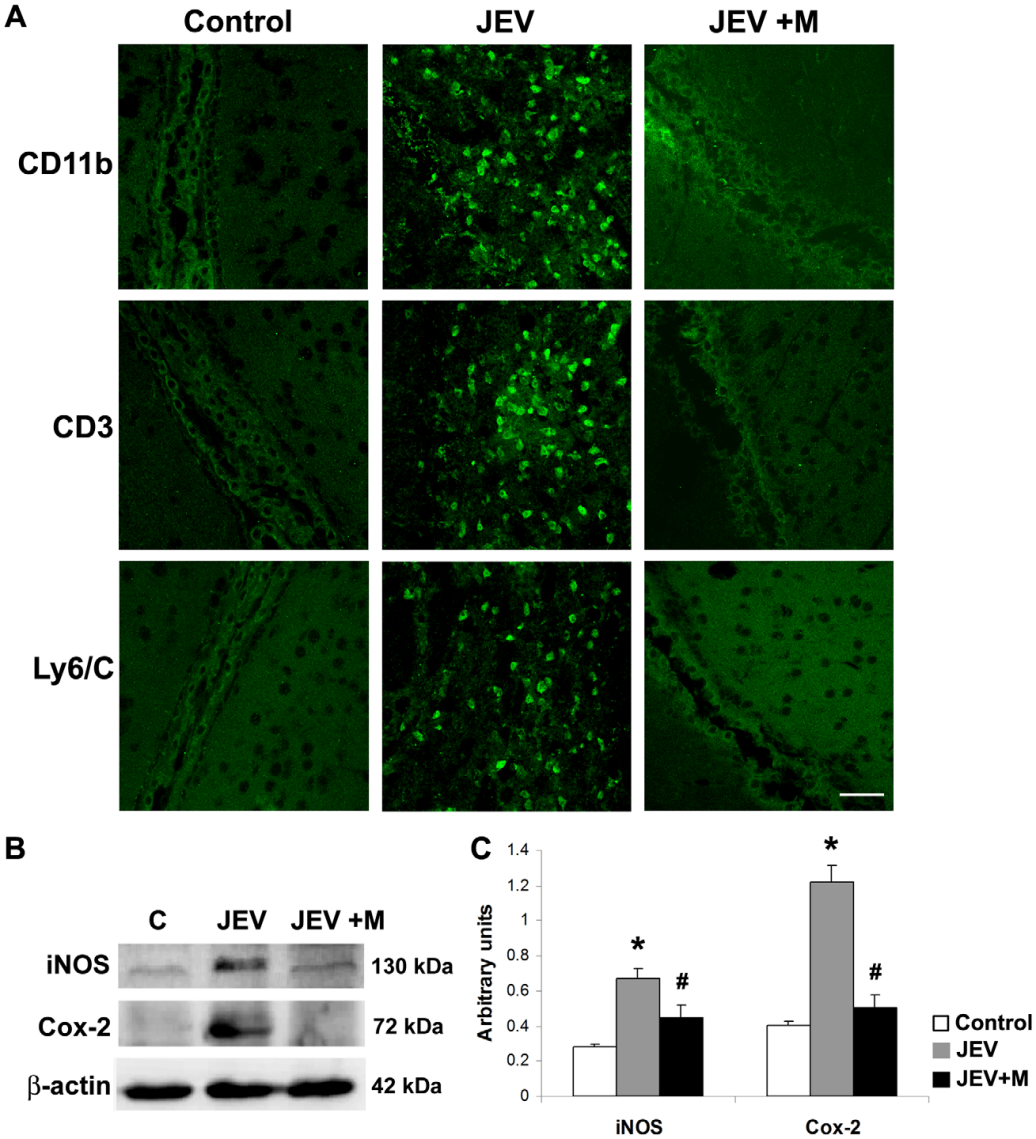
Attenuation of inflammatory milieu in the infected adult SVZ by minocycline administration. Cryostat sections of brains from control, JEV and JEV+M animal groups were stained for cells of monocyte/macrophage lineage (CD11b), T-cells (CD3) and neutrophils (Ly6/C), (FITC, green) (**A**). Images were acquired using oil immersion lens of confocal microscope. These peripheral immune cells were completely absent in the SVZ of control and JEV+M brains. In SVZ of JEV-infected animals, a large number of these cells were observed; scale bar is 50 µ. Protein isolated from SVZ of control, JEV and JEV+M animals were analyzed by immunoblot (**B**). Increased expression of iNOS and Cox-2 in JEV-infected SVZ was reduced significantly in JEV+M SVZ. The graphs represent densitometric quantification of proteins bands of iNOS and Cox-2 normalised to β-actin (**C**). Values represent the means ± SEM from five animals in each group (* significant change from control p<0.01; ^#^ significant change from JEV-infected samples p<0.01).

Since, infiltration of peripheral immune cells is a hallmark of flaviviral infections, we examined the distribution of same immune cells in the JEV-infected SVZ, where already high concentrations of all the cyto/chemokines have been observed. Immunohistochemistry was performed on brain sections from all the 3 groups-C, JEV and JEV+M for different markers of peripheral infiltrating cells. The cells of the monocytic/macrophage lineage including amoeboid microglia are labelled by CD11b and we observed high number of CD11b positive cells in JEV-infected SVZ, but not in control or JEV+M group. The infiltrating CD3 positive T cells were also detected in the SVZ from infected animals, but these cells were completely absent from control and JEV+M animals. Similar distribution was observed for Ly6/c which labels neutrophils, and their infiltration was in quite prominent around the SVZ of JEV animals, but not in SVZ of control or JEV+M animals (Fig. 2A).

In another set of experiments, the protein isolated from SVZ was immunoblotted for the inflammatory mediators iNOS and Cox-2 (Fig. 2B). Increased production of both iNOS and Cox-2 from activated microglia has been observed in JEV-infected pups. In JEV-infected adult SVZ, a significant increase of 2.5-fold in iNOS and 3-fold in Cox-2 expression was observed, compared to control SVZ (Fig. 2B, C) (p<0.01). The enhanced expression of both these inflammatory molecules was decreased significantly in SVZ from JEV+M animals (p<0.01).

### Minocycline treatment to JEV-infected animals results in decrease of infective viral particles in the SVZ

Expression of the viral proteins NS3 and NS5 were found to be significantly decreased in the SVZ of minocycline treated animals post JEV infection as compared to only infected animals (Fig S1A &B) (p<0.01). Plaque assay performed from tissue suspensions from SVZ area, also showed a significant decrease in the number of plaque forming units of the virus following infection and minocycline treatment than only infected group (Fig S1C) (p<0.01).

### Minocycline treatment to JEV-infected animals restores proliferating cells in SVZ

Immunohistochemistry was performed on control, JEV and JEV+M brain sections for the markers of proliferating cells: Ki-67, and PCNA. Also, a set of 3 animals from each group were given a daily dose of BrdU and then stained with anti-BrdU antibody.

**Figure 3:**
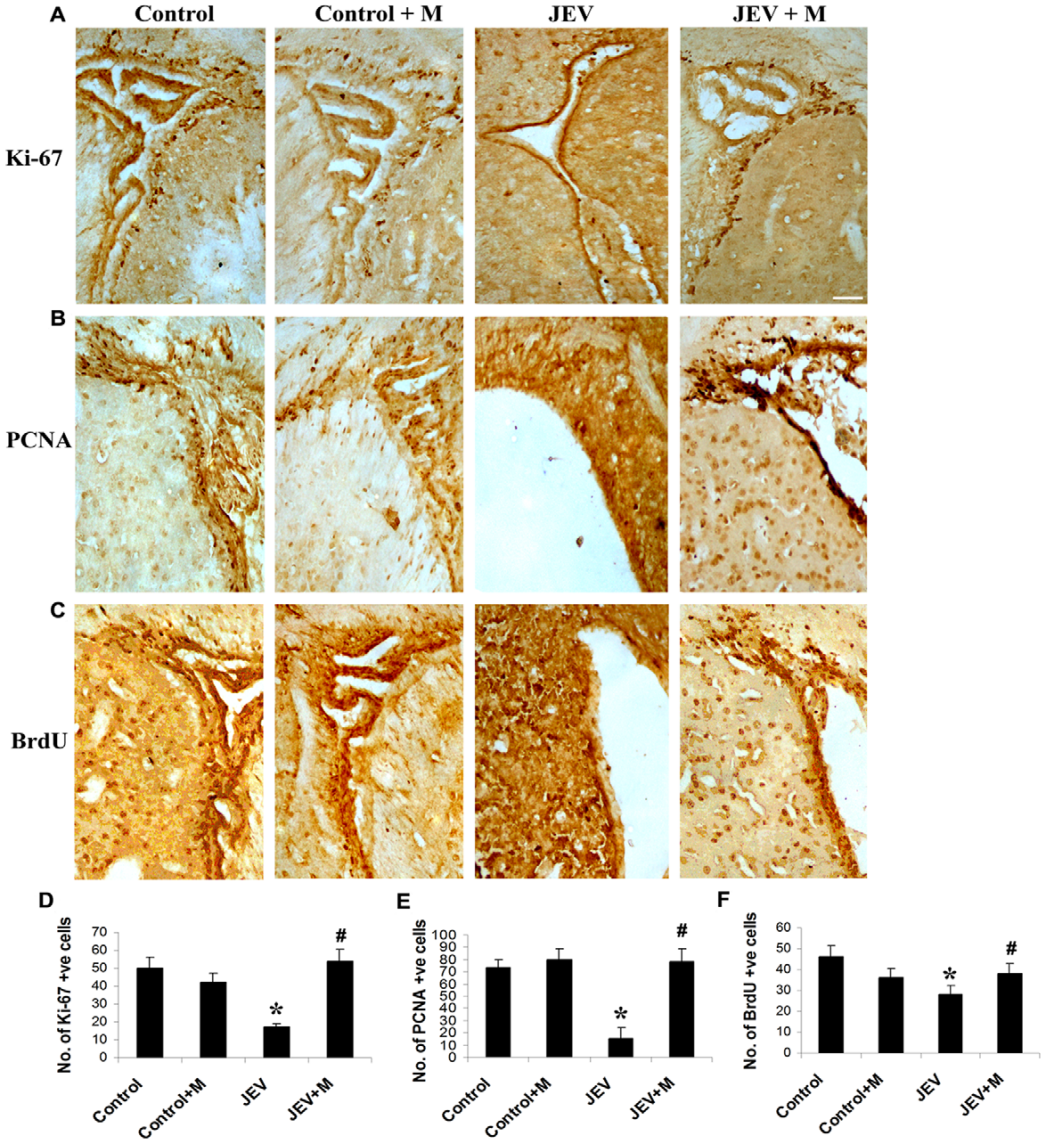
Replenishment of proliferating cells in the infected adult SVZ by minocycline administration. Cryostat sections of brains from control, control+M, JEV and JEV+M animal groups were stained for markers of proliferation- Ki-67 (**A**) and PCNA (**B**) and developed using DAB substrate. BrdU was also administered to animals for 5 days at 50 mg/kg body weight and then animals were sacrificed 6 h after the last BrdU injection. Cryostat sections from BrdU administered animals from all groups were stained using anti-BrdU antibody (**C**). Discrete population of cells in control SVZ show localisation of all the antigens. While reduced population of all three cell types were observed in JEV-infected SVZ, however, they were replenished in the SVZ from JEV+M animals. The graphs represent the number of Ki-67 (**D**), PCNA (**E**) and BrdU (**F**) positive cells in the SVZ from the different treatment groups. Cell counting was done using5 serial sections from 3 animals with Leica IM50 software. Values represent means ± SEM from three animals in each group (* significant change from control, p<0.05; ^#^significant change from JEV-infected animals, p<0.05); scale bar is 50 µ.

Nuclear localisation of both Ki-67 and PCNA was detected in the cells in the SVZ from all the animal groups. Cell counting using Leica IM50 software showed that there is a significant reduction in the number of Ki-67 and PCNA positive cells in the SVZ of JEV-infected animals compared to control animals (Fig. 3A–D) (p<0.05). Minocycline treatment to animals however significantly increased the number of these proliferating Ki-67 and PCNA positive cells over those observed in JEV-infected SVZ (p<0.05). The uptake of BrdU by actively-cycling cells in the SVZ was also determined and a significant 2-fold decrease in their numbers was observed in the infected SVZ compared to control SVZ (Fig. 3E, F) (p<0.05). Moreover, in SVZ of JEV+M animals, the number of BrdU positive cells was significantly higher than those in JEV-infected SVZ (p<0.05), suggesting that minocycline administration to JEV-infected animals restored the pool of cells undergoing cell cycle in the SVZ.

### Minocycline treatment to JEV-infected animals enhances the number of proliferating cells of neuronal lineage in the SVZ

**Figure 4:**
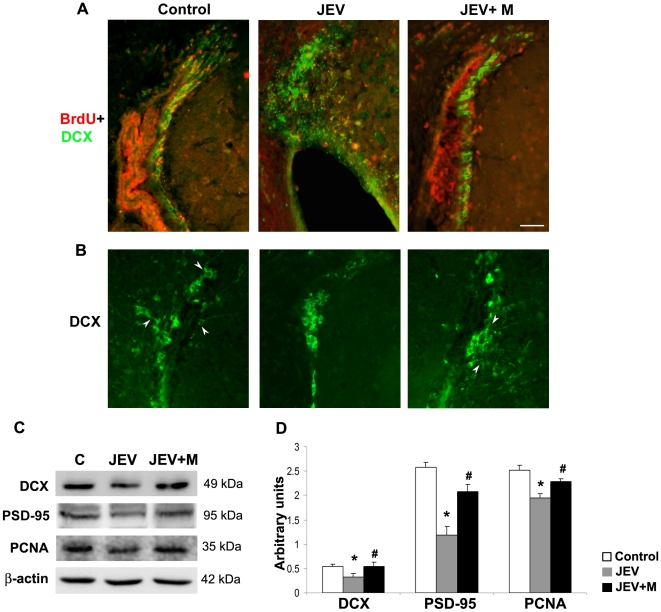
Population of migrating neuroblasts restored in adult infected SVZ by minocycline administration. Cryostat sections of brains from control, JEV and JEV+M animal groups were labelled for proliferating cells of neuronal lineage (BrdU+DCX double staining) (**A**) or for migrating neuroblasts (DCX positive cells) (**B**). Control SVZ show marked co-localization of BrdU (Alexa Fluor, red) and DCX (FITC, green). These double positive cells were distinctly lesser in the JEV-infected SVZ compared to control SVZ, which was however restored in JEV+M SVZ (**A**). DCX staining (FITC, green) show migrating neuroblasts in control SVZ and in SVZ from JEV+M animals with neurite extending outwards (shown by arrowheads), was completely absent in JEV-infected SVZ (**B**); scale bar is 50 µ. Protein isolated from SVZ of control, JEV and JEV+M animals were analyzed by immunoblot (**C**). Expression of DCX, PSD-95 and PCNA declined in JEV-infected SVZ, which in JEV+M SVZ was elevated almost to control levels. The graph represents densitometric quantification of proteins bands of DCX, PSD-95 and PCNA, normalised to β-actin (**D**). Values represent the means ± SEM from five animals in each group (* significant change from control p<0.05; ^#^ significant change from JEV samples p<0.05).

It was important to investigate whether the replenished pool of proliferating cells in the SVZ upon minocycline administration to JEV-infected animals, are of the neuronal lineage and whether they contribute to neurogenesis. We therefore performed double staining for BrdU and DCX to determine the neuronal fate of the BrdU positive cells. A decline in the number of double positive BrdU+DCX cells was observed in the JEV-infected SVZ compared to control SVZ (Fig. 4A). In the JEV+M group however, these BrdU+DCX cells were observed in greater numbers than the infected SVZ. Furthermore, immunohistochemistry for DCX alone showed neurite outgrowth from the migrating neuroblasts in case of control and JEV+M group, but such cells were completely absent from the JEV-infected group (Fig. 4B). This suggests that the migrating neuroblasts in case of control and JEV+M group with neurite protrusions maybe more effective in promoting CNS repair than those cells without the neurite outgrowths.

Quantification of both the proliferating cells and those of the neuronal lineage was done by performing Western blotting for PCNA and DCX respectively. Indeed, a significant decrease in both PCNA and DCX protein expression was observed in JEV-infected SVZ compared to control SVZ. Expression of both these molecules were higher in SVZ from JEV+M group w.r.t JEV-infected group (Fig. 4C, D) (p<0.05). We also checked for expression of PSD-95 which is a post-synaptic density protein, important for formation of functional synapses. A significant 2.5 fold decrease in PSD-95 was observed in JEV-infected SVZ w.r.t control SVZ, probably suggesting the lack of functional synapse formation in infected animals (Fig. 4C, D) (p<0.05). In JEV+M animals, PSD-95 expression was significantly elevated by 2 fold compared to JEV-infected SVZ, implying the functional role of minocycline in CNS regeneration in the infected brain.

### Minocycline attenuates microglial activation following JEV infection *in vitro*

**Figure 5:**
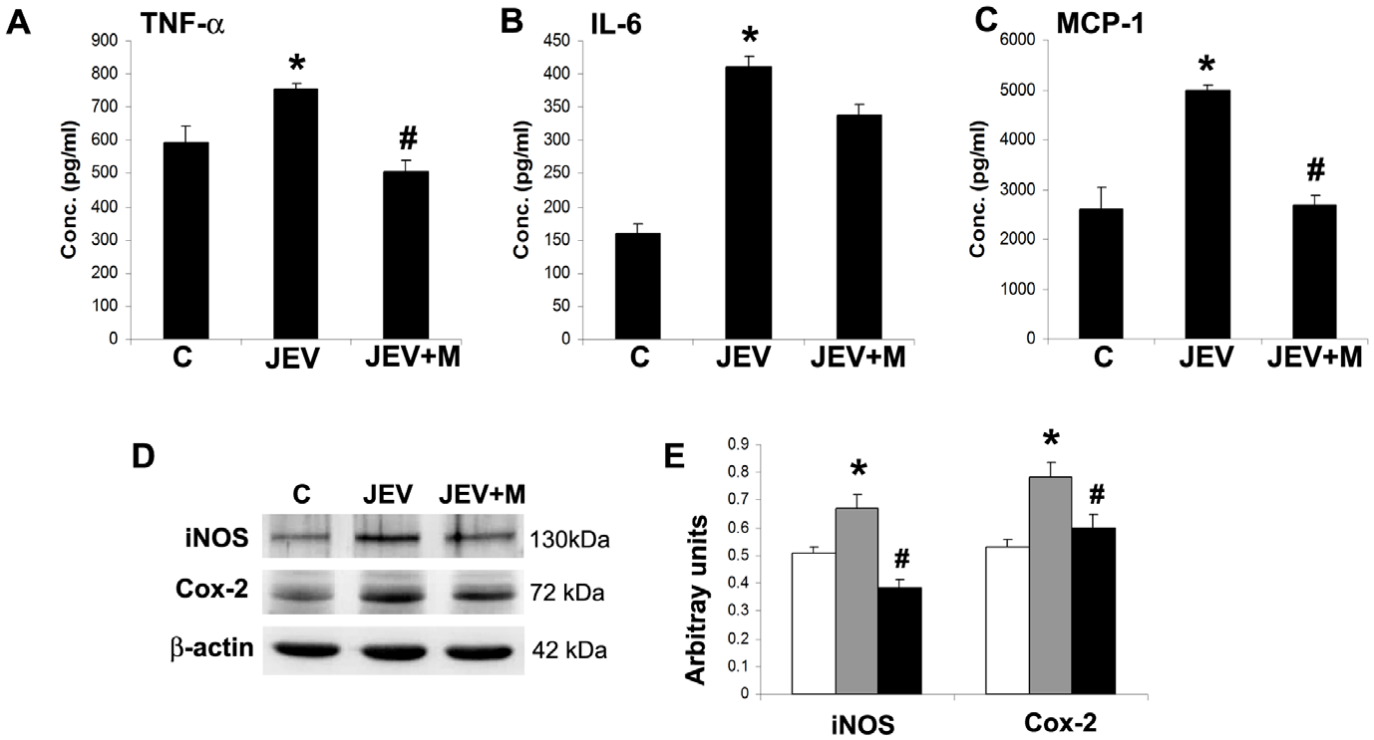
Expression of inflammatory molecules in JEV activated BV2 is attenuated by minocycline treatment. Mouse microglia BV2 were either control (C), JEV-infected (JEV) or JEV-infected and treated with minocycline (JEV+M). After 6 h of minocycline treatment p.i., fresh DMEM-F12 media was added to all conditions. After additional 6 h, CM from BV2 cells from all conditions was collected for CBA and protein extracted for immunoblot. The graphs depict the concentration in pg/ml of cyto/chemokines TNF-α (**A**), IL-6 (**B**) and MCP-1 (**C**) in Control, JEV-infected and JEV+M treated BV2 cells. Values represent means ± SEM from 3 independent experiments performed in duplicate (^*^ significant change from control, p<0.05; ^#^ significant change from JEV, p<0.05). Protein isolated from BV2 cells from C, JEV and JEV+M conditions were analyzed by immunoblot (**D**). The graphs represent the densitometric quantification of protein bands- iNOS and Cox-2 normalised to β-actin (**E**). Values represent mean ± SEM from 3 independent experiments performed in duplicate (^*^ significant change from control, p<0.05; ^#^ significant change from JEV, p<0.05).

Mouse microglia BV2 were either control (C) or JEV-infected (JEV) or infected with JEV and treated with minocycline, both pre and post infection (JEV+M). After 6 h of post-treatment of minocycline, fresh media was added to all the conditions and another 6 h later, the media (CM) were collected for CBA and cell protein were isolated for Western blotting. While the concentration of TNF-α increased significantly in CM from JEV-infected BV2 compared to that from control BV2, however CM from JEV+M cells showed a significant reduction to concentrations below control (Fig. 5A) (p<0.05). Similar elevated concentrations of MCP-1 in CM from JEV-infected BV2 was brought down to control levels in CM from JEV+M treated BV2 (Fig. 5C) (p<0.05). IL-6 concentration was significantly higher in CM from JEV-infected BV2 compared to CM from control cells. Although CM from BV2(JEV+M) showed lower IL-6 concentrations than that from BV2(JEV), however, the difference was not significant (Fig. 5B).

Apart from the cytokines, the expression levels of other inflammatory mediators iNOS and Cox-2 was measured in BV2 cell lysates. Induction of both iNOS and Cox-2 expression was distinct in JEV-infected BV2 compared to control BV2. This increased expression of iNOS and Cox-2 was significantly downregulated by 2 fold in JEV+M treated BV2, compared to JEV-infected BV2 (Fig. 5D, E) (p<0.05).

### Soluble inflammatory mediators from JEV-activated microglia inhibit proliferation of NSPCs, which is reversed by minocycline treatment

**Figure 6:**
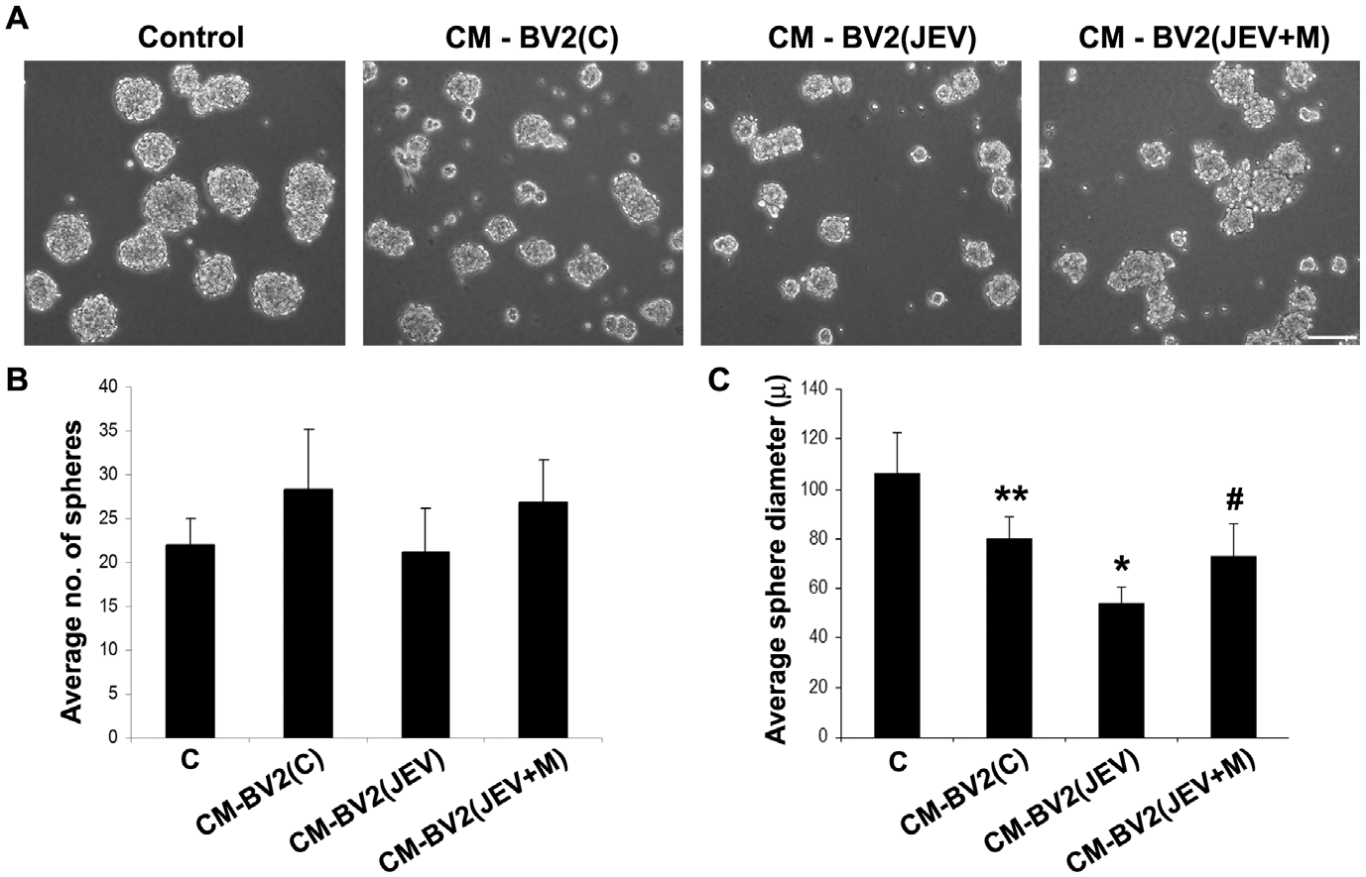
Impaired neurosphere formation by soluble mediators from JEV activated microglia is reversed by minocycline treatment. Single cell suspensions of NSPCs were cultured alone (C) or in presence of BV2-derived CM under 3 conditions- BV2(C), BV2(JEV), BV2(JEV+M) for 3 days. Phase-contrast micrographs of neurospheres were acquired under 10× magnification (**A**). Scale bar is 100 µ. The number of neurospheres was counted from 10 different fields for each condition and the average diameter of the spheres was measured using Leica IM50 software. The graphs represent the average number of spheres (**B**) and their average diameter in microns (**C**). The numbers of colony forming neurospheres do not show any significant difference between groups (**B**). The reduction in size of neurospheres grown in CM from BV2(JEV) was prominent compared to CM-BV2(C) or CM-BV2(JEV+M) conditions (**C**). Values represent mean ± SEM from 3 independent experiments performed in duplicate (^**^ significant change from control, p<0.05; ^*^ significant change from CM-BV2(C), p<0.01; ^#^ significant change from CM-BV2(JEV), p<0.01).

In order to determine the extent to which the soluble factors released from JEV activated microglia directly affect NSPC development, the cells were grown in CM obtained from BV2 cells. After two passages, single cell suspensions of NSPCs were grown for 3 days either alone (C), or in presence of 50% of CM from BV2 cells in 3 conditions- BV2(C), BV2(JEV) and BV2(JEV+M). The numbers of neurospheres formed after 3 days in culture as well as their average diameter (both indicators of their proliferative capacity) were calculated using Leica IM50 software (Fig. 6). No significant differences in the number of spheres generated were observed between the different conditions (Fig. 6A, B). However, the average sphere diameter showed a significant decrease when NSPCs were grown in CM from BV2(JEV) compared to that from BV2(C) or control neurospheres (Fig. 6A, C) (p<0.01). NSPCs grown in presence of CM from BV2(JEV+M) however resulted in more number of spheres and with average diameter greater than those grown in BV2(JEV) media (p<0.01).

**Figure 7:**
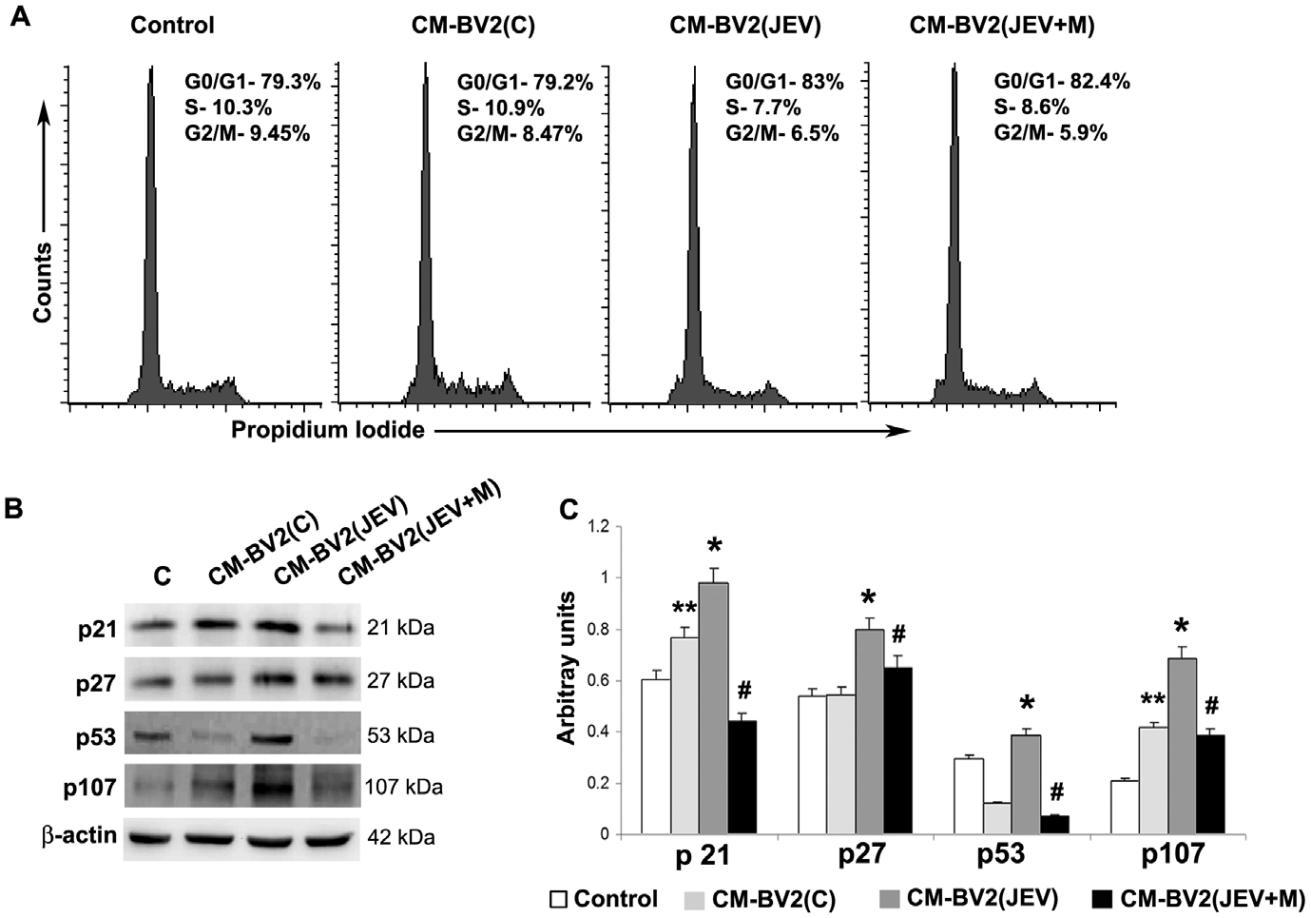
Induction of cell cycle arrest by soluble mediators from JEV activated microglia is reversed by minocycline treatment. Cell cycle analysis of single cell suspensions of neurospheres grown alone or in presence of BV2-CM was performed using RNase/PI buffer and detected by FACS. Gates were set to assess the percentages of G0/G1 (2n DNA), S (>2n DNA) and G2/M (4n DNA) cells using Cell Quest Pro Software, and the representative histograms have been shown (**A**). Data is representative of 3 independent experiments. Protein isolated from control and BV2-CM treated neurospheres were immunoblotted for checkpoint proteins p21, p27, p53 and p107 (**B**). The expression of all these proteins increased in neurospheres grown in CM from BV2(JEV) compared to that from BV2(C) or from BV2(JEV+M) (**C**). Densitometric analysis of immunoblot normalised to β-actin was plotted as a bar graph. Values represent mean ± SEM from three independent experiments. (^**^ significant change from control, p<0.05; * significant change from CM-BV2(C), p<0.05; ^#^ significant change from CM-BV2(JEV), p<0.05).

In another set of experiments, NSPCs grown alone or in presence of BV2-derived CM was collected after 3 days for cell cycle analysis. Single cell suspensions of NSPCs were stained with PI, and acquired in FACS Calibur. Following gating of specific population, the percentages of cells lying in the different stages of cell cycle (G0/G1, S and G2/M phase) were analysed using Cell Quest Pro software. In case of control neurospheres, approximately 10% of cells were in the S-phase, which was comparable to the NSPCs grown in CM from BV2(C) (Fig. 7A). However, the percentage of cells in S-phase decreased significantly to 7.7% when grown in CM from BV2(JEV), and was restored back to 8.7% in presence of CM from BV2(JEV+M). Interestingly, as the percentages of S-phase NSPCs declined in presence of BV2(JEV), more cells were observed in the G0/G1 phase (Fig. 7A). This possibly indicates a G1→S phase arrest in these NSPCs when grown in CM from BV2(JEV) compared to that from BV2(C), and the arrest was partially unblocked when CM from BV2(JEV+M) was used. These are indications that the soluble inflammatory mediators from JEV-activated microglia have anti-proliferative effect on NSPCs, which is partially recovered by minocycline treatment to BV2 cells due to reduced inflammatory molecules production.

Considering that the CM from activated microglia modulates G1→S phase transition, we determined the expression of cell cycle checkpoint proteins by western blotting from cell lysates of NSPCs grown in different CM. The expression of p21, p27, p53 and p107 was determined, all of which act during progression of cells from G1→S stage. Both p21 and p27 are specific checkpoint proteins for G1→S phase transition and are significantly up-regulated in NSPCs grown in CM from BV2(JEV) compared to CM from BV2(C) (Fig. 7B, C) (p<0.05). Similar increase in expression was observed for p53 and p107 (both Retinoblastoma proteins) in case of NSPCs grown in CM from BV2(JEV) (p<0.05). Interestingly, all these checkpoint proteins were significantly down-regulated in NSPCs when CM from BV2(JEV+M) was used compared to that from BV2(JEV) (Fig. 7B, C) (p<0.05). Taken together, these findings clearly indicate that soluble inflammatory mediators released from JEV-activated BV2 cells blocks G1→S phase cell cycle transition via upregulation of various checkpoint proteins- an effect which is reversed using soluble mediators from JEV-infected and minocycline treated BV2 cells.

### Soluble inflammatory mediators from JEV-activated microglia inhibit neuronal differentiation of NSPCs, which is reversed by minocycline treatment

**Figure 8:**
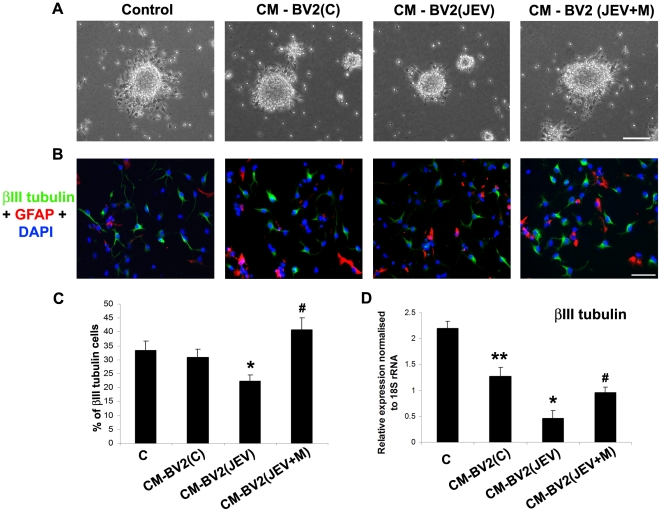
Decreased neuronal differentiation of NSPCs by soluble mediators from JEV activated microglia is reversed by minocycline treatment. Phase contrast micrographs of differentiating neurospheres from control and BV2 derived CM treated NSPCs 2 days after induction of differentiation on PDL-coated plates (**A**). Impaired migration and differentiation of cells from the periphery of the spheres is observed in CM-BV2(JEV) conditions. Control and BV2-Cm treated neurospheres were dissociated and single cell suspensions of NSPCs were differentiated for 2 days. The cells were fixed and double immunocytochemistry for β-III tubulin (neuronal phenotype) and GFAP (astrocytic phenotype) was performed and mounted with Vectashield containing DAPI. Distinct β-III tubulin positive cells (FITC, green) and GFAP positive cells (Alexa 594, red) were observed, indicating NSPCs undergoing differentiation (**B**). The percentage of β-III tubulin positive cells were calculated from 5 different fields and plotted as a representative graph (**C**). RNA was isolated from these NSPCs after 2 days of differentiation and qRT-PCR was performed for β-III tubulin expression. The graph represents relative mRNA expression of β-III tubulin normalised to the expression of constitutive 18S rRNA (**D**). Values represent mean ± SEM from three independent experiments performed in duplicate. (^**^ significant change from control, p<0.05; ^*^ significant change from CM-BV2(C), p<0.01; ^#^ significant change from CM-BV2(JEV), p<0.01). Scale bar corresponds to 50 microns.

Neurospheres grown alone or in presence of CM from BV2 cells for 3 days were plated on PDL-coated plates and allowed to differentiate for 2 days. Micrographs of adhered neurospheres show the migration of the cells from the periphery of the spheres and differentiation into neurons (Fig. 8A). After induction of differentiation, while neurons with elongated neurites extended noticeably from the periphery of the spheres in control and in CM-BV2(C) treated, lesser proportion of differentiating cells was observed in CM from BV2(JEV) treated neurospheres. Neurospheres grown in CM from BV2(JEV+M) however showed considerable proportion of differentiating cells emerging from the sphere periphery (Fig. 8A).

In a simultaneous experiment, neurospheres from the different conditions were dissociated into single cell suspensions and then allowed to differentiate for 2 days on PDL-coated chamber slides. Immunocytochemistry revealed the presence of β-III tubulin positive cells indicative of neuronal differentiation and GFAP positive cells indicative of astrocytic differentiation. The percentages of these newly formed neurons (β-III tubulin) and astrocytes (GFAP) were calculated for each condition. Differentiation of CM-BV2 (JEV) treated NSPCs yielded significantly fewer numbers of neurons than the NSPCs from CM of BV2(C) or control conditions (Fig. 8B, C) (p<0.01). Moreover, a 2 fold increase in the percentage of β-III tubulin cells was observed in NSPCs from CM of BV2(JEV+M) as compared to that of BV2(JEV) (p<0.01). Additionally, we also assessed for the transcript levels of β-III tubulin in these differentiating NSPCs by qRT-PCR. A significant decrease in β-III tubulin expression in differentiated NSPCs from CM of BV2(JEV) was observed compared to CM from BV2(C) or from BV2(JEV+M) (Fig. 8D) (p<0.01). Overall, the neurospheres/NSPCs grown in CM from JEV-activated microglia exhibit impaired neuronal differentiation even when the CM was removed during the differentiation regime, thereby suggesting that the exposure to soluble mediators from microglia for 3 days modulated the developmental pattern of these NSPCs. To extend further, neurospheres/NSPCs grown in CM from BV2(JEV+M) differentiate normally, possibly due to the reduction of anti-neurogenic inflammatory mediators in the BV2-derived CM by minocycline treatment.

## Discussion

As the primary immune effector cells of the CNS, microglia responds to injury or to the presence of pathogens by becoming activated. Upon activation, microglia undergoes proliferation, chemotaxis, and morphological alterations and generates numerous mediators involved in the inflammatory and immunomodulatory response. The ensuing neuroinflammation have a myriad of effects- from clearing the viral pathogen, to remodelling brain parenchyma, as well as modulating neuronal health and neurogenesis by the long-range diffusible soluble mediators and the local inflammatory milieu.

Our previous studies have already established how JEV infection in adult BALB/c mice leads to an inflammatory upheaval in the adult CNS [30]. In this study, we observed that there is differential distribution of the various cyto/chemokines in the different areas of the brain; the most affected areas being the cortex and the neurogenic niche of SVZ. Minocycline, a second-generation tetracycline derived antibiotic has been proven as an effective measure of attenuating JEV-induced microglial activation, as well as alleviating the clinical symptoms of JE [30]. This present study further corroborated that minocycline administration to adult animals following JEV infection dramatically lowered the level of cytokines in all the investigated brain areas and therefore promoted animal survivability. The response to cerebral JEV infection is characterized at the pathological level by significant recruitment and extravasation of inflammatory cells [36], [37], which is, partly mediated by chemokines released from the activated microglia [38]. Though such infiltration of peripheral immune cells was prominent in the infected SVZ, minocycline treated animals showed barely any of these peripheral immune infiltrates. This partly results from the ability of minocycline to reduce production of inflammatory molecules and chemokines which signal to the peripheral immune cells as well as their ability to maintain the integrity of BBB in JEV infections [30], [39]. Besides the cyto/chemokines, the expression of other inflammatory molecules iNOS and Cox-2 was elevated in JEV-infected SVZ but reduced significantly following minocycline treatment. Taken together, these findings clearly established that inflammatory milieu created in the SVZ during JEV infection is completely alleviated by minocycline treatment.

The central role of inflammation in modulating neurogenesis is still debatable, and often dependent on the disease model, and the duration and intensity of the inflammatory response. One of the best characterised neurogenic niches is SVZ [40], the endogenous brain compartment housing the self-renewing NSPCs, which have immense potential of tissue repair upon CNS injury [41], [42]. Functional impairment of the SVZ germinal niche has been shown following either cranial irradiation [43], or LPS-mediated acute inflammation [44] or EAE-induced persistent inflammation [45]. Inflammation associated with ischemic insults and stroke compromise the survivability of the newly formed striatal neurons that are generated from the SVZ after stroke [46], [47]. Our investigation also revealed a significant reduction in the actively replicating cells including those belonging to the neuronal lineage in the JEV-infected SVZ niche. Administration of the anti-inflammatory compound minocycline was able to restore the NSPC population back to that in control SVZ. Minocycline readily crosses the BBB and suppress microglial activation in a number of neurodegenerative disorders as well as virus infections [30], [48]. Similarly, in models of LPS inflammation and stroke, minocycline decreased microglial activation, accounting for the potentiation of NSPC pool and neurogenesis [14], [28]. However, no direct effect of minocycline in stimulating endogenous neurogenesis in control animals has been reported, which is corroborated by our *in vivo* findings. Minocycline administration to control animals did not result in any significant increase in the number of proliferating cells in the neurogenic niche, i.e. the SVZ. Hence, it is plausible that minocycline has no direct effect on neurogenesis. Inflammatory blockade usually is accompanied by a broad spectrum of effects which promote neurogenesis, like reduction of cell death of new born neurons [14], attenuation of HPA axis activation [49], or the decrease in pro-inflammatory cytokines and serum glucocorticoid levels [15], [50].

Our *in vitro* studies provided further evidence that the detrimental effects of JEV-induced microglial activation on the development of NSPCs is via release of soluble inflammatory mediators from the activated microglia, which is reversed upon minocycline treatment. Upregulation of various inflammatory markers iNOS and Cox-2 indicated the activation of mouse microglial cells BV2 by JEV, which was significantly attenuated with minocycline treatment to BV2 cells. The stress signaling kinases were also elevated upon JEV infection in BV2, which was considerably reduced by minocycline treatment (data not shown). CM from JEV-activated BV2 cells was enriched with pro-inflammatory mediators and cyto/chemokines, and mouse neurospheres grown in this CM showed impaired proliferation and neuronal differentiation. This was corroborated by previous findings where CM from LPS-activated BV2 cells inhibited the formation of new neurons (DCX^+^) and led to apoptosis [15]. Acutely activated microglia, or their CM, reduced NSPC survival, prevented neuronal differentiation and strongly increased glial differentiation, likely through the release of pro-inflammatory cytokines, whereas chronically activated microglia promoted both neuronal/glial differentiation and cell survival of NSPCs [51]. Pro-inflammatory cytokine TNF-α contributes to apoptotic death of hippocampal progenitor cells [52] and mouse SVZ derived neurospheres [53]. Although low concentrations of TNF-α stimulate NSPC proliferation [54], and are regarded as pro-neurogenic [55], however, TNF-α has also been shown to inhibit proliferation of striatal NSPCs [16]. Microglia-derived IL-6 is a potent anti-neurogenic cytokine which promotes astrocytic differentiation of NSPCs at the expense of neuronal differentiation [15], [56]. The physiological role of iNOS/NO as a negative regulator of SVZ neurogenesis [57], [58] as well as an inhibitor of neurosphere proliferation by blocking EGF-receptor and PI3K/Akt pathway has been established [59]. NO exposure to adult NSPCs inhibits neurogenesis by decreasing expression of the proneural gene Ngn-2 and β-III tubulin, and is instead beneficial for astroglial differentiation [60]. Interestingly, CM from BV2(JEV+M) rescued both the proliferative and differentiation potential of these NSPCs, thereby indicating the restorative effect of anti-inflammatory minocycline treatment on neurogenesis. Other p38MAPK inhibitor with potency to inhibit microglial activation, have been shown to be neuroprotective and promote neurogenesis in hypoxic hippocampal slice cultures [61].

We have further elucidated that the inhibition of NSPC proliferation by CM from BV2 results from a blockade of cell cycle progression mainly at G1→S phase due to the upregulation of various checkpoint proteins. The retinoblastoma proteins p53 and p107 are up-regulated which in turn induces other checkpoint proteins, like p21 and p27. All these proteins together act on the cyclin/CDK complexes, and inhibit their functions, thereby inducing cell cycle arrest. NSPCs grown in CM from BV2(JEV+M) however, exhibited low expression of these checkpoint kinases, which allows them to cycle normally. Induction of quiescence in NSPCs by chemokines has been reported via upregulation of p21 and p27 [22].

Microglial activation and CNS inflammation have been associated with cognitive impairment in a number of brain disorders like Lewy Body dementia, AD, and AIDS dementia. Cranial irradiation also leads to inflammation in the SGZ and progressive cognitive deterioration. A link between onset of AIDS dementia complex in HIV-individuals to compromised neurogenesis as a consequence of uncontrolled inflammatory processes has been proposed [62], [63]. Therefore, the use of anti-inflammatory compounds and NSAIDs has shown promise in improving the cognitive attributes in inflammatory neurodegenerative disorders and in virus-induced inflammation. Since we observed no direct effect of minocycline on neurogenesis, our findings highlights its role in reduction of the inflammatory milieu in the SVZ germinal niche and resultant rescue NSPCs from the toxic effects of the inflammatory mediators. The restoration of SVZ neurogenesis by minocycline administration in JEV-infected animals may have important implications in promoting brain's regenerative capacity following neuronal loss in JE. The use of minocycline in a wide variety of neurological disorders has been reported [64]. Minocycline has also been proposed as a potential therapeutic measure which can possibly reduce HIV-induced cognitive problems [65] and human trials for such are in progress (http://clinicaltrials.gov/ct2/show/NCT00855062). From our study we see that besides exerting neuroprotective effects, minocycline, by virtue of its anti-inflammatory properties, stimulate neurogenesis in JEV infection, thereby reasserting its immense therapeutic potential in treatment of JE.

## Supporting Information

Figure S1 **Decreased viral protein expression and infective virl particle production following minocycline treatment.** Immunoblot showing significantly decreased expression of viral NS3 and NS5 proteins in SVZ of infected mice treated with minocycline (**A**). Densitometric analysis of immunoblot normalised to β-actin was plotted as a bar graph. Values represent mean ± SEM from three independent experiments (**B**). A significant decrease in the number of infective viral particle formation was also observed in the SVZ of minocycline treated infected animals when compared to non-minocycline treated animals. Values are mean ± SEM from 2 independent experiments (**C**). (^*^ significantly increased in JEV-infected as compared to control, p<0.01; ^#^ significantly decreased following minocycline treatment as compared to only JEV-infected, p<0.01).
(TIF)
