# Supplementary figures and images for: Abrogated Inflammatory Response Promotes Neurogenesis in a Murine Model of Japanese Encephalitis
Source: PLoS One. 2011 Mar 3;6(3):e17225. doi: 10.1371/journal.pone.0017225 (PMC3048396; doi:10.1371/journal.pone.0017225)

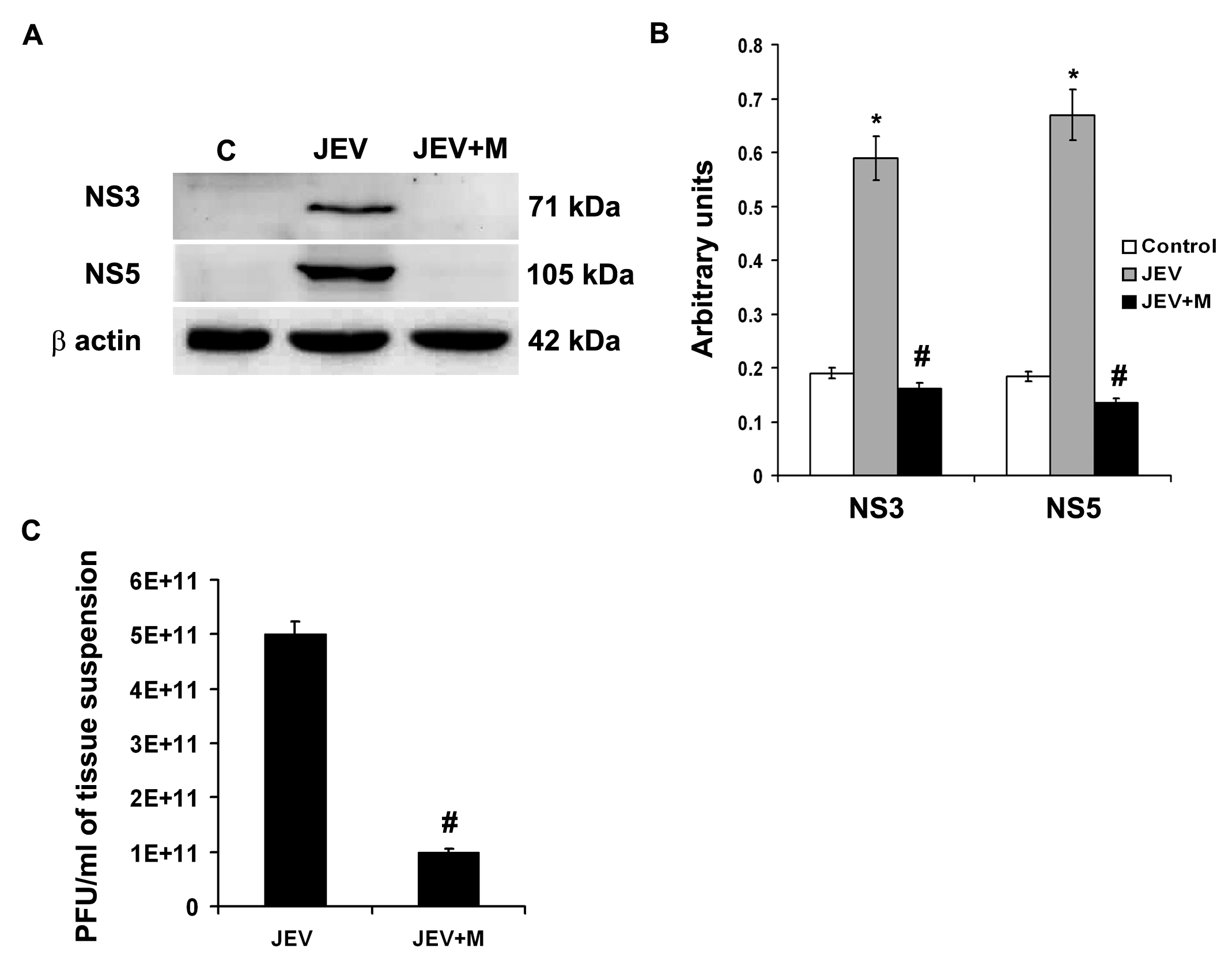

Supplement: Figure S1 — Decreased viral protein expression and infective virl particle production following minocycline treatment. Immunoblot showing significantly decreased expression of viral NS3 and NS5 proteins in SVZ of infected mice treated with minocycline (A). Densitometric analysis of immunoblot normalised to β-actin was plotted as a bar graph. Values represent mean ± SEM from three independent experiments (B). A significant decrease in the number of infective viral particle formation was also observed in the SVZ of minocycline treated infected animals when compared to non-minocycline treated animals. Values are mean ± SEM from 2 independent experiments (C). (* significantly increased in JEV-infected as compared to control, p<0.01; # significantly decreased following minocycline treatment as compared to only JEV-infected, p<0.01). (TIF) [file pone.0017225.s001.tif]
